# Percentages of Surgical Procedure Combinations That Were Performed Just Once or Twice at Florida Hospital and Ambulatory Surgery Centers During Each Quarter From 2010 Through 2024

**DOI:** 10.7759/cureus.103338

**Published:** 2026-02-10

**Authors:** Franklin Dexter, Brenda G Fahy, Richard H Epstein

**Affiliations:** 1 Anesthesia, University of Iowa, Iowa City, USA; 2 Anesthesiology, University of Florida, Gainesville, USA; 3 Anesthesiology, Miller School of Medicine, University of Miami, Miami, USA

**Keywords:** ambulatory surgical procedures, appointments and schedules, bayesian methods, current procedural terminology, elective surgical procedures, international classification of diseases, machine learning, operating rooms, operative time

## Abstract

Introduction: Data-intensive machine learning is suitable for predicting the case durations of common surgical procedures. In contrast, Bayesian methods (e.g., applying the surgeon/scheduler’s estimate) often perform well for uncommon procedures. Uncommon procedures dramatically affect case duration predictions, necessary for scheduling cases days to weeks before surgery. Procedure coding systems have changed over time, as has their disparate use, due to the large increase in ambulatory surgery. However, the current epidemiology of uncommon procedures is based on datasets from 25 years ago. We calculated contemporaneous incidence proportions for rare procedure combinations, those performed at facilities only once or twice per quarter.

Methods: The retrospective cohort study used de-identified, publicly available data from the 2010-2024 Florida ambulatory surgery databases, comprising distinct combinations of 20,014,189 cases distributed across 5,106,524 combinations of quarter, facility, and Current Procedural Terminology codes. There were also 11,643,813 cases in the 2009-2024 inpatient databases across 4,772,566 combinations of quarter, facility, and distinct combinations of International Classification of Diseases (ICD) procedure codes.

Results: The incidence proportions of procedures performed once or twice at each facility during the quarter performed, “doubletons,” became progressively less common. In contrast, the change from ICD-9 to the more granular ICD-10-PCS in 2015 made singletons and doubletons more common for inpatient surgery. In 2024, approximately 66%, 78%, and 87% of procedures were observed just once or twice each quarter at the ambulatory surgery center, hospital outpatient department, and inpatient surgical suite where observed. These doubleton procedures accounted for approximately 18% of cases at ambulatory surgery centers, 36% at hospital outpatient departments, and 55% at inpatient surgical suites. Pooling hospital estimates, approximately 84% of procedures and approximately 44% of cases were among procedures performed just once or twice during the quarter.

Conclusions: Although surgical procedure(s) are the most important predictors of operating room time, many procedures are performed rarely, resulting in little historical case duration data for case duration estimation. Freestanding ambulatory surgery centers have, for more than 10 years, remained different from hospitals in performing more common procedures. Hospitals should plan, when scheduling cases, that approximately 84% of distinct combinations of procedures and 44% of cases will have little to no procedure-specific historical data, and even less so by the combination of procedure and surgeon. Machine learning models for predicting case durations should therefore account for these uncommon procedures, for which Bayesian methods are well suited.

## Introduction

Over the past five years, more than 100 articles have been published describing models for predicting surgical case duration, many in recent review articles [1,2]. While it may seem intuitive that using machine learning with many variables known at the time a case is scheduled to achieve smaller mean absolute errors in operating room time would lead to improved operating room and anesthesia productivity (more cases performed per unit time), this has been found to be false for several reasons [3-7]. One reason is that unless facilities incorporate a predictive method that includes a Bayesian component relying on the surgeon’s and scheduler’s prior judgment (e.g., their estimate), most variability in case durations contributing to lower productivity and reduced productivity of surgeons from late starts is caused by cases of uncommon procedures [6,7]. A systematic review of comparisons between machine learning and traditional methods for case duration prediction reported no studies that used an endpoint of productivity or the efficiency of use of operating room or anesthesia time [2].

Potentially, the importance of using Bayesian methods to address uncommon procedures may have decreased over time, with fewer facilities having many rare single surgical procedures or combinations of surgical procedures performed during cases [8,9]. For convenience, we henceforth refer to these categories simply as "procedures." For example, the incidence proportions of rare procedures may have changed due to improvements in procedure coding systems, the closure of smaller hospitals, and differential referral patterns to ambulatory surgery centers versus inpatient surgical suites. The contemporary generalizability of the managerial epidemiology of rare procedures is unknown because the studies that drove scientific development in case duration prediction were conducted using datasets that are now >25 years old [10-12].

Our primary objective was to estimate the incidence proportions of procedures that were performed only once or twice at the facility in the state of Florida during the quarter of 2024 when and where the procedure was performed [12,13]. We refer to those procedures performed only once as "singletons" and, for convenience, those occurring once or twice as "doubletons." Operating room and anesthesia times depend highly on the specific surgeon performing the procedure at each facility [14,15]. If a procedure was performed just once or twice at a facility during a quarter, the combination of procedure and surgeon will also be a singleton or doubleton.

Our secondary objective was to examine longitudinal changes in the incidence proportions of rare procedures. We hypothesized that, for inpatient procedures, the incidence of rare procedures would be higher currently (2016-2024) than from 2009 to 2015 because of the change from International Classification of Diseases version 9 (ICD-9) to ICD-10 Procedure Coding Systems [8]. The reason for this conjecture is that ICD-10 is more granular, with many thousands more codes [8]. We hypothesized that, between 2010 and 2024, there would be changes in the incidence of singletons and doubletons at freestanding ambulatory surgery centers and hospital outpatient departments due to dynamic health system changes. However, we lacked insight into whether these changes would result in higher or lower incidence proportions.

## Materials and methods

The Institutional Review Board of the University of Miami determined that this retrospective analysis of de-identified, publicly available data does not constitute human subjects research. We utilized publicly available records from the Florida Agency for Health Care Administration (AHCA; Tallahassee, FL). As a matter of policy, AHCA disclaims responsibility for the results and conclusions of studies for which it provides data. Our analyses relied on knowing surgical "cases," not discharges, and thus needed the dates of the individual procedures [16]. Following approval of a second data use agreement with AHCA, the regular records were supplemented with the date of each encounter (for ambulatory patients) and the date of each hospital admission (for inpatients) [17,18]. However, these dates were not used for analysis; they were only used to group cases into the year-and-quarter category used in our analyses. We performed the calculations through 2024 because that was the most recent full year of data available at the time of analysis.

The ambulatory surgery database, 2010-2024, contained 5,106,524 combinations of facility, quarter, and procedure (Table 1). We started with 2010 for ambulatory surgery because that was the first year that state reporting of ambulatory surgery was mandated for all facilities in Florida. The facility type was selected from the database using the field named PROCODE as follows: freestanding ambulatory surgery center or hospital outpatient department. The combinations of Current Procedural Terminology (CPT) codes for each date and patient were combined in alphabetical sequence. The codes included were broadly defined as surgery, encompassing major therapeutic, major diagnostic, and minor therapeutic procedures. Codes excluded were those without American Society of Anesthesiologists’ Relative Value Guide base units, thereby limiting consideration to those commonly performed with an anesthesia practitioner. Historically, ambulatory surgery was limited to procedures with seven or fewer base units [19,20]. Therefore, we repeated our primary calculations after excluding the few cases with more than seven base units (Table 2). Procedures with more than seven base units are physiologically complex [20,21].

**Table 1:** Characteristics of the Current Procedural Terminology ambulatory surgery data reported as n (%) of the combinations of quarter, facility, and procedure(s). While Table 1 shows counts of procedures, Table 2 shows counts of the corresponding cases, categorized by procedure. The n’s in the header are the denominator for each column. The ambulatory surgery centers accounted for 39.2% of the total 5,106,524 combinations of quarter, facility, and procedure. Everywhere in the article except for the fourth through eighth rows, "procedure" refers to the distinct combination of procedures (i.e., sequence is not considered).

Characteristics	Ambulatory surgery centers (n = 2,002,603)	Hospital outpatient departments (n = 3,103,921)	Combined (n = 5,106,524)
Singleton, a procedure combination with only one case during the quarter at the facility, n (%)	1,257,589 (62.8)	2,059,736 (66.4)	3,317,325 (65.0)
Doubleton, procedure combinations with one or two cases during the quarter at the facility, n (%)	1,524,628 (76.1)	2,469,372 (79.6)	3,994,000 (78.2)
Combinations with more than seven American Society of Anesthesiologists’ base units, n (%)	42,005 (2.1)	102,647 (3.3)	144,652 (2.8)
One procedure in the combination, n (%)	1,128,204 (56.3)	1,872,869 (60.3)	3,001,073 (58.8)
Two procedures in the combination, n (%)	611,059 (30.5)	902,074 (29.1)	1,513,133 (29.6)
Three procedures in the combination, n (%)	193,788 (9.7)	250,792 (8.1)	444,580 (8.7)
Four or more procedures in the combination, n (%)	69,552 (3.5)	78,186 (2.5)	147,738 (2.9)
2010 was the year, n (%)	132,587 (6.6)	197,109 (6.4)	329,696 (6.5)
2011 was the year, n (%)	130,935 (6.5)	201,739 (6.5)	332,674 (6.5)
2012 was the year, n (%)	131,174 (6.6)	205,509 (6.6)	336,683 (6.6)
2013 was the year, n (%)	132,131 (6.6)	208,330 (6.7)	340,461 (6.7)
2014 was the year, n (%)	129,543 (6.5)	207,672 (6.7)	337,215 (6.6)
2015 was the year, n (%)	130,685 (6.5)	212,799 (6.9)	343,484 (6.7)
2016 was the year, n (%)	130,316 (6.5)	213,023 (6.9)	343,339 (6.7)
2017 was the year, n (%)	129,285 (6.5)	209,569 (6.8)	338,854 (6.6)
2018 was the year, n (%)	130,843 (6.5)	210,767 (6.8)	341,610 (6.7)
2019 was the year, n (%)	137,334 (6.9)	210,183 (6.8)	347,517 (6.8)
2020 was the year, n (%)	118,973 (5.9)	166,149 (5.4)	285,122 (5.6)
2021 was the year, n (%)	143,510 (7.2)	214,276 (6.9)	357,786 (7.0)
2022 was the year, n (%)	142,136 (7.1)	217,747 (7.0)	359,883 (7.0)
2023 was the year, n (%)	142,564 (7.1)	215,124 (6.9)	357,688 (7.0)
2024 was the year, n (%)	140,587 (7.0)	213,925 (6.9)	354,512 (6.9)

**Table 2:** Characteristics of the Current Procedural Terminology ambulatory surgery data reported as n (%) of the cases by combination of quarter, facility, and procedure(s). While Table 1 displays the counts of procedures, this matching Table 2 shows the counts of the corresponding cases, categorized by procedure. The n’s in the header are the denominator for each column. The ambulatory surgery centers accounted for 55.4% of the total 20,014,189 cases. Everywhere in the article except for the fourth through eighth rows, "procedure" refers to the distinct combination of procedures.

Characteristics	Ambulatory surgery centers (n = 11,081,391)	Hospital outpatient departments (n = 8,932,798)	Combined (n = 20,014,189)
Singleton, cases of a procedure combination with only one case during the quarter at the facility, n (%)	1,257,589 (11.3)	2,059,736 (23.1)	3,317,325 (16.6)
Doubleton, cases of procedure combinations with one or two cases during the quarter at the facility, n (%)	1,791,667 (16.2)	2,879,008 (32.2)	4,670,675 (23.3)
Combinations with more than seven American Society of Anesthesiologists’ base units, n (%)	221,556 (2.0)	450,072 (5.0)	671,628 (3.4)
One procedure in the combination, n (%)	9,377,217 (84.6)	7,065,234 (79.1)	16,442,451 (82.2)
Two procedures in the combination, n (%)	1,311,152 (11.8)	1,466,596 (16.4)	2,777,748 (13.9)
Three procedures in the combination, n (%)	296,477 (2.7)	313,881 (3.5)	610,358 (3.0)
Four or more procedures in the combination, n (%)	96,545 (0.9)	87,087 (1.0)	183,632 (0.9)
2010 was the year, n (%)	639,414 (5.8)	547,811 (6.1)	1,187,225 (5.9)
2011 was the year, n (%)	632,505 (5.7)	550,292 (6.2)	1,182,797 (5.9)
2012 was the year, n (%)	658,953 (5.9)	556,659 (6.2)	1,215,612 (6.1)
2013 was the year, n (%)	681,682 (6.2)	564,175 (6.3)	1,245,857 (6.2)
2014 was the year, n (%)	686,094 (6.2)	561,169 (6.3)	1,247,263 (6.2)
2015 was the year, n (%)	701,014 (6.3)	582,788 (6.5)	1,283,802 (6.4)
2016 was the year, n (%)	729,731 (6.6)	591,458 (6.6)	1,321,189 (6.6)
2017 was the year, n (%)	735,671 (6.6)	583,047 (6.5)	1,318,718 (6.6)
2018 was the year, n (%)	757,976 (6.8)	595,322 (6.7)	1,353,298 (6.8)
2019 was the year, n (%)	783,030 (7.1)	602,753 (6.7)	1,385,783 (6.9)
2020 was the year, n (%)	624,856 (5.6)	461,749 (5.2)	1,086,605 (5.4)
2021 was the year, n (%)	829,426 (7.5)	646,998 (7.2)	1,476,424 (7.4)
2022 was the year, n (%)	845,827 (7.6)	683,861 (7.7)	1,529,688 (7.6)
2023 was the year, n (%)	884,984 (8.0)	703,398 (7.9)	1,588,382 (7.9)
2024 was the year, n (%)	890,228 (8.0)	701,318 (7.9)	1,591,546 (8.0)

For the inpatient database, 2009-2024, we limited ICD-9-PCS and ICD-10-PCS codes to those related to major therapeutic or major diagnostic procedures. We included 2009 because inpatient reporting was already required, and we had encounter dates for that year. The combinations of ICD-9-PCS or ICD-10-PCS codes were sorted alphabetically for each case. The ICD-10-PCS includes laterality, which does not affect case duration for many procedures (e.g., right versus left hip arthroplasty) [22]. Therefore, before calculating each combination, anatomic sites with a symmetric organ (e.g., left arm versus right arm) were changed to the left-sided procedure code, as previously described [22]. The admission date for each patient was combined with the number of days after admission when the procedure(s) were performed in the database to provide the date each combination of procedures was performed.

Our primary goal was to estimate incidence proportions for 2024. We did so for four dependent variables. First, there was the incidence proportion of procedures at a facility during a quarter for which the procedure was observed only once during the quarter at the facility. These are “singletons.” Because we limited our consideration to observed procedures, all combinations were observed at least once [12]. As explained above, all consideration of “procedure” refers to the distinct combination of procedures, excluding considerations of laterality, as our focus is the prediction of case durations [22]. Second, there was the incidence proportion of procedures at a facility during a quarter, for which the procedure was observed once or twice during that quarter. As explained in the previous sections, we refer to this dependent variable as "doubletons." Third, there were instances of cases that were procedures that were singletons. Fourth, some cases were of procedures that were doubletons.

Conceptually, estimates of precision were unnecessary because we used every surgical case performed at every hospital and ambulatory surgery center in the state from 2010 through 2024 (i.e., no sample was taken). Nevertheless, we calculated 99% confidence intervals for incidence proportions as estimates of precision for three reasons. First, precision for making comparisons with other states and provinces depends not only on the total numbers of procedures and cases, but also on the variability of estimates among facilities [23]. Therefore, future investigators could not infer our standard errors from the raw counts provided. Second, the variability among facilities is quantified as the variance on the logit scale, which is not directly interpretable on the incidence proportion scale [23]. Simple summary measures of variability calculated on the incidence proportion scale would be biased estimates of this heterogeneity and could not be accurately transformed by future investigators into the logit scale variance they would need. Third, for some organizations, single facility codes can represent multiple buildings on a campus, potentially leading to considerable variability across facilities [24]. For these reasons, we relied on mixed-effects logistic regression to estimate the precision of the incidence proportions, while incorporating the variability among facilities as a random effect [23]. For modeling the ambulatory surgery database, the following two binary fixed effects were used: (a) ambulatory surgery center or hospital outpatient department, and (b) 2023 or 2024. We used the last two years of data to estimate 2024, increasing the sample size while using a categorical model for the year to avoid modeling changes over time. For the study of the inpatient surgery database, the only fixed effect was year (2023 or 2024).

Each of the four primary dependent variables was summarized twice, both the conditional and marginal means of the incidence proportions [23]. The conditional mean estimates of incidence refer to the average facility's incidence proportions. These were calculated using Stata version 19.5 (College Station, TX: StataCorp.) with the melogit command, followed by nlcom to take the inverse logit of the linear sum of the parameter estimates. The marginal mean was the estimate calculated across all facilities, weighted by the frequencies for each facility. We calculated the marginal mean by running melogit with 18 integration points and then using the margins command for the year 2024 [23].

As explained in the previous section, our secondary goal was to evaluate changes over the years in the observed incidence proportions of singletons and doubletons. We used Spearman's rank correlation coefficient to do so. There were eight combinations examined as follows: (a) hospital outpatient departments or ambulatory surgery centers, (b) singletons or doubletons, and (c) procedures or cases. For the eight comparisons, two-sided p-values were reported after Bonferroni adjustment, with p<0.05 after adjustment considered statistically significant. The exact p‑values were calculated using Monte Carlo simulations with 10,000 permutations, using the Stata spearman command. For the inpatient database, we knew a priori that there would be more procedures by ICD-10-PCS than by ICD-9-PCS, but not by how much [8,9]. Therefore, we did not test that association inferentially; instead, we displayed it graphically.

## Results

Tables 1, 2 show the characteristics of the 20,014,189 cases in the ambulatory surgery database distributed among 5,106,524 combinations of quarter, facility, and combinations of Current Procedural Terminology codes. Table 1 presents the data by procedure, and Table 2 presents the data by case. Tables 3, 4 show the characteristics of the 11,643,813 cases in the inpatient surgery database distributed among 4,772,566 combinations of quarter, facility, and combinations of International Classification of Diseases version 9 and 10 Procedure Coding System codes. The most common outpatient procedure was extracapsular cataract removal with intraocular lens insertion. The most common inpatient procedure was low cervical cesarean delivery. Despite common procedures, the overall ratios of total cases to total combinations of quarter, facility, and procedure were only 3.9 for ambulatory surgery (20,014,189/5,106,524) and 2.4 for inpatient surgery (11,643,813/4,772,566).

**Table 3:** Characteristics of the International Classifications of Diseases inpatient surgery data reported as n (%) of the combinations of quarter, facility, and procedure(s). While Table 3 shows counts of procedures, Table 4 shows counts of the corresponding cases, categorized by procedure. The n’s in the header are the denominator for each column. Everywhere in the article except for the third through 13th rows, "procedure" refers to the distinct combination of procedures (i.e., sequence is not considered).

Characteristics	ICD-9 Clinical Modification (n = 1,715,956)	ICD-10 Procedure Coding System (n = 3,056,610)	Combined (n = 4,772,566)
Singletons, a procedure combination with only one case during the quarter at the facility, n (%)	1,121,416 (65.4)	2,177,233 (71.2)	3,298,649 (69.1)
Doubletons, procedure combinations with one or two cases during the quarter at the facility, n (%)	1,422,654 (82.9)	2,682,684 (87.8)	4,105,338 (86.0)
One procedure in the combination, n (%)	613,991 (35.8)	1,086,790 (35.6)	1,700,781 (35.6)
Two procedures in the combination, n (%)	559,289 (32.6)	897,092 (29.3)	1,456,381 (30.5)
Three procedures in the combination, n (%)	291,172 (17.0)	474,007 (15.5)	765,179 (16.0)
Four procedures in the combination, n (%)	138,855 (8.1)	268,299 (8.8)	407,154 (8.5)
Five procedures in the combination, n (%)	60,351 (3.5)	140,183 (4.6)	200,534 (4.2)
Six procedures in the combination, n (%)	25,337 (1.5)	78,514 (2.6)	103,851 (2.2)
Seven procedures in the combination, n (%)	11,726 (0.7)	43,030 (1.4)	54,756 (1.1)
Eight procedures in the combination, n (%)	5,948 (0.3)	25,541 (0.8)	31,489 (0.7)
Nine procedures in the combination, n (%)	3,547 (0.2)	15,380 (0.5)	18,927 (0.4)
10 procedures in the combination, n (%)	2,243 (0.1)	9,423 (0.3)	11,666 (0.2)
11 or more procedures in the combination, n (%)	3,497 (0.2)	18,351 (0.6)	21,848 (0.5)
2009 was the year, n (%)	256,027 (14.9)	0 (0.0)	256,027 (5.4)
2010 was the year, n (%)	255,587 (14.9)	0 (0.0)	255,587 (5.4)
2011 was the year, n (%)	253,736 (14.8)	0 (0.0)	253,736 (5.3)
2012 was the year, n (%)	252,408 (14.7)	0 (0.0)	252,408 (5.3)
2013 was the year, n (%)	250,275 (14.6)	0 (0.0)	250,275 (5.2)
2014 was the year, n (%)	251,606 (14.7)	0 (0.0)	251,606 (5.3)
2015 was the year, n (%)	196,317 (11.4)	83,063 (2.7)	279,380 (5.9)
2016 was the year, n (%)	0 (0.0)	332,219 (10.9)	332,219 (7.0)
2017 was the year, n (%)	0 (0.0)	332,050 (10.9)	332,050 (7.0)
2018 was the year, n (%)	0 (0.0)	333,358 (10.9)	333,358 (7.0)
2019 was the year, n (%)	0 (0.0)	334,676 (10.9)	334,676 (7.0)
2020 was the year, n (%)	0 (0.0)	315,194 (10.3)	315,194 (6.6)
2021 was the year, n (%)	0 (0.0)	318,132 (10.4)	318,132 (6.7)
2022 was the year, n (%)	0 (0.0)	326,590 (10.7)	326,590 (6.8)
2023 was the year, n (%)	0 (0.0)	338,663 (11.1)	338,663 (7.1)
2024 was the year, n (%)	0 (0.0)	342,665 (11.2)	342,665 (7.2)

**Table 4:** Characteristics of the International Classifications of Diseases inpatient surgery data reported as n (%) of the cases by combinations of quarter, facility, and procedure(s). While Table 3 displays the counts of procedures, this matching Table 4 shows the counts of the corresponding cases, categorized by procedure. The n’s in the header are the denominator for each column. Everywhere in the article except for the third through 13th rows, "procedure" refers to the distinct combination of procedures (i.e., sequence is not considered).

Characteristics	ICD-9 Clinical Modification (n = 4,989,551)	ICD-10 Procedure Coding System (n = 6,654,262)	Combined (n = 11,643,813)
Singletons, cases of procedure combinations with only one case during the quarter at the facility, n (%)	1,121,416 (22.5)	2,177,233 (32.7)	3,298,649 (28.3)
Doubletons, cases of procedure combinations with one or two cases during the quarter at the facility, n (%)	1,723,892 (34.6)	3,188,135 (47.9)	4,912,027 (42.2)
One procedure in the combination, n (%)	3,133,274 (62.8)	3,726,569 (56.0)	6,859,843 (58.9)
Two procedures in the combination, n (%)	1,017,563 (20.4)	1,357,866 (20.4)	2,375,429 (20.4)
Three procedures in the combination, n (%)	439,938 (8.8)	650,428 (9.8)	1,090,366 (9.4)
Four procedures in the combination, n (%)	205,802 (4.1)	371,257 (5.6)	577,059 (5.0)
Five procedures in the combination, n (%)	90,301 (1.8)	201,686 (3.0)	291,987 (2.5)
Six procedures in the combination, n (%)	42,178 (0.8)	121,754 (1.8)	163,932 (1.4)
Seven procedures in the combination, n (%)	22,341 (0.4)	72,904 (1.1)	95,245 (0.8)
Eight procedures in the combination, n (%)	12,869 (0.3)	47,499 (0.7)	60,368 (0.5)
Nine procedures in the combination, n (%)	8,230 (0.2)	31,151 (0.5)	39,381 (0.3)
10 procedures in the combination, n (%)	5,439 (0.1)	20,970 (0.3)	26,409 (0.2)
11 or more procedures in the combination, n (%)	11,616 (0.2)	52,178 (0.8)	63,794 (0.5)
2009 was the year, n (%)	757,519 (15.2)	0 (0.0)	757,519 (6.5)
2010 was the year, n (%)	749,913 (15.0)	0 (0.0)	749,913 (6.4)
2011 was the year, n (%)	735,406 (14.7)	0 (0.0)	735,406 (6.3)
2012 was the year, n (%)	728,792 (14.6)	0 (0.0)	728,792 (6.3)
2013 was the year, n (%)	723,735 (14.5)	0 (0.0)	723,735 (6.2)
2014 was the year, n (%)	730,612 (14.6)	0 (0.0)	730,612 (6.3)
2015 was the year, n (%)	563,574 (11.3)	181,727 (2.7)	745,301 (6.4)
2016 was the year, n (%)	0 (0.0)	739,065 (11.1)	739,065 (6.3)
2017 was the year, n (%)	0 (0.0)	740,821 (11.1)	740,821 (6.4)
2018 was the year, n (%)	0 (0.0)	737,520 (11.1)	737,520 (6.3)
2019 was the year, n (%)	0 (0.0)	743,421 (11.2)	743,421 (6.4)
2020 was the year, n (%)	0 (0.0)	673,034 (10.1)	673,034 (5.8)
2021 was the year, n (%)	0 (0.0)	680,838 (10.2)	680,838 (5.8)
2022 was the year, n (%)	0 (0.0)	699,448 (10.5)	699,448 (6.0)
2023 was the year, n (%)	0 (0.0)	726,141 (10.9)	726,141 (6.2)
2024 was the year, n (%)	0 (0.0)	732,247 (11.0)	732,247 (6.3)

**Figure 1:**
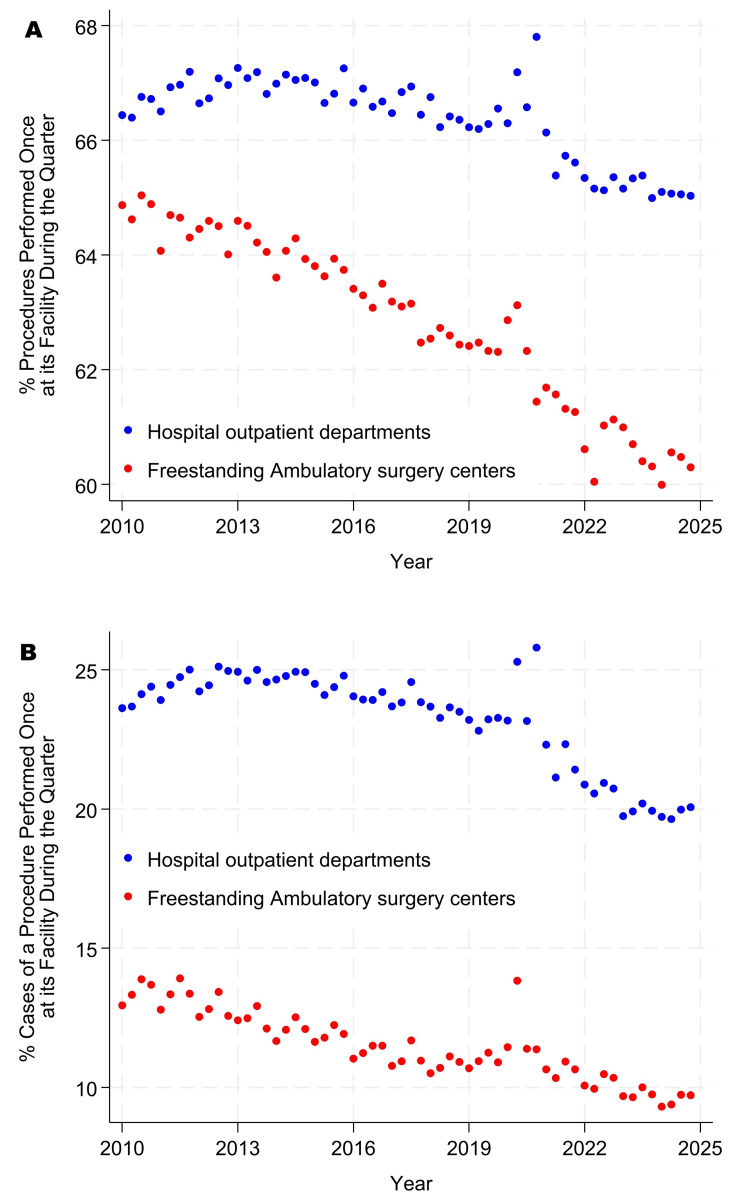
Percentages of procedures that were singletons in the ambulatory surgery database. Singletons were observed only once during the quarter at a given facility. Panel A presents counts by procedure, as in Table 1, whereas panel B presents counts by case, as in Table 2. A procedure is defined as a distinct combination of Current Procedural Terminology (CPT) codes. For example, cases comprising a breast lumpectomy (CPT: 19301) and excision of deep axillary lymph nodes (CPT: 38525) were treated as one distinct procedure when counting cases performed at each facility and quarter. From top to bottom, Spearman rank correlations with year were -0.72, -0.98, -0.76, and -0.90, respectively, all with Bonferroni-adjusted exact p<0.0001. The third quarter of 2020 corresponds to the COVID-19 pandemic shutdown period.

**Figure 2:**
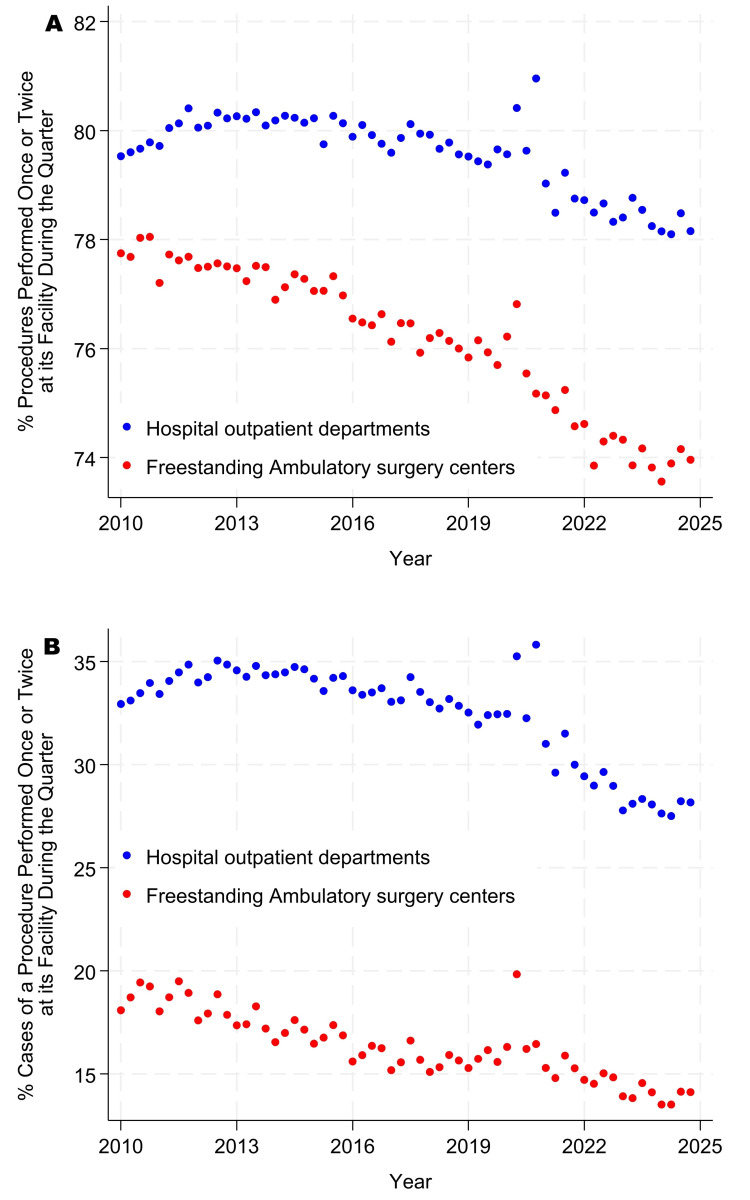
Percentages of procedures that were singletons or doubletons in the ambulatory surgery database. Panel A presents counts by procedure, as in Table 1, whereas panel B presents counts by case, as in Table 2. Singletons were observed only once during the quarter at a given facility, and doubletons were observed once or twice. A procedure is defined as a distinct combination of Current Procedural Terminology (CPT) codes. From top to bottom, Spearman rank correlations with year were -0.69, -0.80, -0.75, and -0.88, all with Bonferroni-adjusted exact p<0.0001. The third quarter of 2020 corresponds to the COVID-19 pandemic shutdown period.

**Figure 3:**
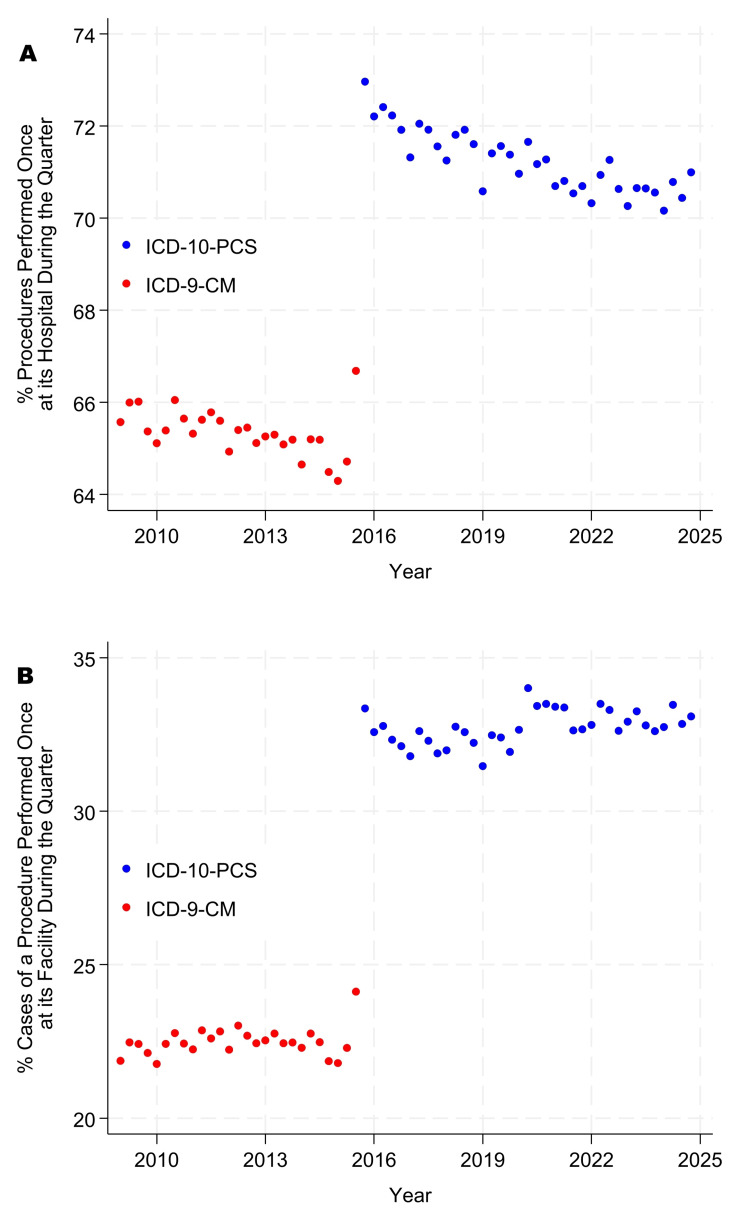
Percentages of procedures that were singletons in the inpatient database. Singletons were observed only once during the quarter at a given facility. Panel A presents counts by procedure, as in Table 3, whereas panel B presents counts by case, as in Table 4. A procedure is defined as a distinct combination of International Classification of Diseases (ICD) procedure codes. For example, cases comprising reposition of the tibia (0QSH04Z) and fibula (0QSK04Z) with internal fixation were treated as one distinct procedure for the purpose of counting cases at each hospital and quarter. Discharges prior to October 2015 were coded using ICD-9, and those thereafter using ICD-10. For ICD-10 procedures, before calculating each combination, anatomic sites involving symmetric organs (e.g., left versus right arm) were recoded to the left-sided procedure code to remove the effect of laterality [22].

**Figure 4:**
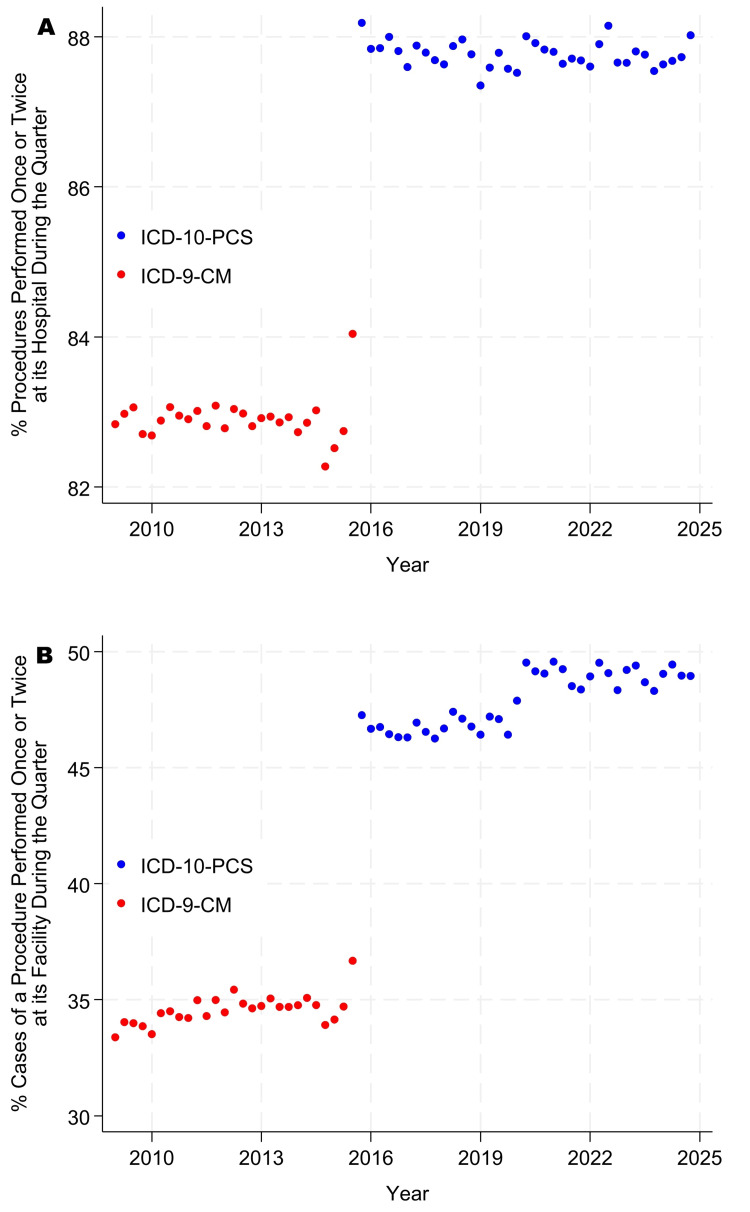
Percentages of cases involving procedures that were singletons or doubletons in the inpatient database. Panel A presents counts by procedure, as in Table 3, whereas panel B presents counts by case, as in Table 4. Singletons were observed only once during the quarter at a given facility, and doubletons were observed once or twice during the quarter at the same facility. A procedure is defined as a distinct combination of International Classification of Diseases (ICD) codes. Discharges prior to October 2015 were coded using ICD-9, and those thereafter using ICD-10. For ICD-10 procedures, before calculating each combination, anatomic sites involving symmetric organs (e.g., left versus right arm) were recoded to the left-sided procedure code to remove the effect of laterality [22].

The incidence proportions of singletons and doubletons became progressively less common from 2010 through 2024 (Figure 1 panels A, B, and Figure 2 panels A, B). In contrast, the use of ICD-10-PCS made singletons and doubletons more common for inpatient surgery than when ICD-9-PCS was used (Figure 3 panels A, B, and Figure 4 panels A, B). Table 5, Figure 1 panels A, B, and Figure 3 panels A, B show that, while controlling for heterogeneity among facilities, in 2024, procedures were performed just once or twice each quarter at their facility for approximately 66% of procedures at ambulatory surgery centers, 78% at hospital outpatient departments, and 87% at inpatient surgical suites. From Table 5, Figure 2 panels A, B, and Figure 4 panels A, B, these doubleton procedures accounted for approximately 18% of cases at ambulatory surgery centers, 36% at hospital outpatient departments, and 55% at inpatient surgical suites. Results for ambulatory surgery differed little when repeated, excluding physiologically complex procedures (Table 5). Pooling the estimates across hospitals, approximately 84% of hospitals’ procedures would be doubletons (78.1% × 3,103,921 + 87.1% × 4,772,566)/(3,103,921 + 4,772,566), and approximately 44% of cases (36.3% × 8,932,798 + 55.3% × 6,654,262)/(8,932,798 + 6,654,262).

**Table 5:** Estimated conditional and marginal means of the incidence proportions for 2024, the primary results for ambulatory surgery centers, hospital outpatient departments, and inpatient surgical suites. "Singleton" means the procedure or combination of procedures was performed only once during the quarter at the facility. "Doubleton" means the procedure or combination of procedures was performed only once or twice during the quarter at the facility. The first and second columns are conditional mean estimates of the incidence percentages. The third and fourth columns present marginal mean estimates of incidence percentages (i.e., pooled across all facilities). Both were estimated using mixed-effects logistic regression, with the facility serving as the random effect. "ASC" represents an ambulatory surgery center. "HOD" represents the hospital outpatient department. There is a row with the exclusion of seven or more base units, representing the exclusion of cases with more than seven American Society of Anesthesiologists’ Relative Value Guide base units (i.e., physiologically complex procedures typically associated with inpatient surgical care).

Variables	Estimate of the mean of the average facility	99% confidence interval	Marginal (weighted) mean estimate	99% confidence interval
Singletons at ASCs (%)	53.2	51.7-54.7	53.1	51.6-54.5
Singletons at HODs (%)	66.2	64.4-68.0	65.5	63.7-67.3
Doubletons at ASCs (%)	67.5	65.9-69.0	66.5	64.9-68.0
Doubletons at HODs (%)	79.4	77.8-81.0	78.1	76.5-79.7
Cases of procedures that were singletons and performed at ASCs (%)	7.78	6.73-8.82	11.9	10.4-13.3
Cases of procedures that were singletons and performed at HODs (%)	23.1	19.4-26.7	27.9	24.5-31.3
Cases of procedures that were doubletons and performed at ASCs (%)	12.6	10.9-14.2	17.7	15.8-19.6
Cases of procedures that were doubletons and performed at HODs (%)	32.6	27.9-37.3	36.3	32.4-40.2
Singletons at ASCs, excluding >7 base units (%)	53.5	51.9-55.1	53.3	51.8-54.8
Singletons at HODs, excluding >7 base units (%)	66.9	65.0-68.8	66.1	64.3-68.0
Singletons or doubletons at ASCs, excluding >7 base units (%)	67.9	66.2-69.5	66.7	65.2-68.3
Singletons or doubletons at HODs, excluding >7 base units (%)	80.2	78.6-81.8	78.8	77.1-80.4
Cases of procedures that were singletons and performed at ASCs, excluding >7 base units (%)	7.90	6.82-8.98	12.2	10.7-13.6
Cases of procedures that were singletons and performed at HODs, excluding >7 base units (%)	24.5	20.6-28.4	29.3	25.8-32.8
Cases of procedures that were doubletons and performed at ASCs, excluding >7 base units (%)	12.7	11.1-14.5	18.1	16.2-20.1
Cases of procedures that were doubletons and performed at HODs, excluding >7 base units (%)	34.6	29.7-39.5	38.0	34.0-42.0
Singletons among inpatient cases (%)	71.4	69.8-73.0	70.5	69.0-72.1
Doubletons among inpatient cases (%)	88.1	87.2-89.0	87.1	86.1-88.1
Cases of procedures that were singletons and performed as inpatient surgery (%)	37.2	34.6-39.9	38.4	35.9-41.0
Cases of procedures that were doubletons and performed as inpatient surgery (%)	56.1	52.8-59.4	55.3	52.5-58.2

## Discussion

Earlier, investigators sought to understand the relative contributors to variability in operating room schedules and case duration predictions, specifically the influences on the tardiness of starts for to-follow surgeons [3,5-8,11-15]. "Tardiness" is zero minutes if the patient enters the operating room on or before the scheduled start time; otherwise, it is the number of minutes entered after the scheduled time. The managerial epidemiological studies found that most procedures were performed infrequently, and many cases involved these uncommon procedures [8-13]. These results were important because they showed that data-intensive machine learning methods alone yield little to no increase in operating room or anesthesia group productivity [3,5]. For example, reducing the mean absolute error by 50% without any other managerial changes resulted in a 1.0% decrease in the number of cases performed per room per day [3]. In contrast, Bayesian methods rely on shared prior knowledge (e.g., the surgeon and scheduler’s estimate) [6,7]. Probabilistic Bayesian methods facilitate filling holes in the schedule without increasing overutilized time, thereby increasing productivity [6]. The problem we addressed in this article is that incidence proportions for uncommon procedures can change over time, and the earlier managerial epidemiological studies are now decades old [10-12].

As hypothesized in the previous sections, we observed changes over the years in the incidence proportions of procedures performed just once ("singletons") or once or twice ("doubletons") at their facility each quarter. Statewide, approximately 44% of hospital cases were doubletons. By design, that finding vastly underestimates the problem in practice, because surgeons with the first case of the day start [25], often perform more than one case on days when operating [26,27], causing a geometric increase in the probability of at least one of those multiple cases lacking historical data [28]. For example, with just two such cases, 2 × 44% - 44% equals 69% of lists having at least one such case with little or no historical data [2]; nonetheless, for the specific surgeon, as needed to estimate case durations accurately [14,15]. The time required for a list of cases cannot be accurately estimated before the cases start, when there are no historical values for individual cases, and if secondary information related to task duration is not incorporated [29].

Integration of clinical estimates (Bayesian methods) continues to be needed

Bayesian methods compensate for procedures with limited historical data (e.g., by mathematically weighting the surgeon and scheduler’s estimate with available historical operating room data) [30,31]. For decisions involving the advance scheduling of a case into an operating room, regression analyses can be used when there is one or two scheduled procedures for the case [32]. Variables included the median time estimate for each component procedure, type of anesthesia, urgent status, patient age, and the specialties of the component procedures [32]. Alternatively, for this decision and the many others made in operating room management before cases begin (e.g., the longest reasonable time for case completion), Bayesian methods can be used to combine historical data with the surgeon or scheduler estimate [30,33]. In the Bayesian estimation process, the value of the surgeon or scheduler estimate can be quantified as the equivalent number of historical cases, represented by the weighting parameter, τ [34]. This framework yields a graduated, relative contribution of the surgeon or scheduler estimate and the historical data [7,33,34]. For example, when the value of the surgeon or scheduler estimate is τ = 3.0 historical cases, and 12 or more historical durations are available for the scheduled procedure, the surgeon or scheduler estimate has a small (20%) effect on the calculated duration; 3.0/(12 + 3.0) = 0.2 [34,35]. In contrast, when there is just one historical case for the same procedure, the surgeon/scheduler estimate makes a larger (75%) contribution; 3.0/(1 + 3.0) = 0.75 [34,35]. The variability around the surgeon/scheduler’s estimated case duration can be calculated separately for each service or pooled across all services [6,7,30].

Simple grouping of similar procedures has not addressed the problem of limited historical data

Using groups of codes or textual inference from procedure names to increase sample size has been an insufficient approach for case scheduling to reduce to-follow surgeons’ mean tardiness of starts, because different procedures take different amounts of time [6,36]. Procedure pooling increases root mean squared predictive errors and coefficients of variation [36]. Meanwhile, increases in sample size alone cause negligible reductions in the mean tardiness of case starts [37]. At hospitals, increasing the historical sample size from one to 39 only reduced mean tardiness by 4 min [37]. Therefore, if recognized, the issue of observing singletons and doubletons is not that rare procedures cause increased mean tardiness, but rather the mathematical consequence that they generally prevent automated data-only machine learning from being an effective solution for advance scheduling (e.g., provide predictions for less than half the lists of cases). However, if unrecognized, the consequences are large because for procedures that are singletons or doubletons, machine learning estimates alone are conceptually analogous to Sample Average Approximations, relying on those few available historical case durations to estimate future operating room times. Relying on such approximations resulted in approximately 25.38% higher costs due to overutilized and underutilized time caused by suboptimal case sequencing [38]. Unless incorporating a predictive method that includes a Bayesian component relying on the surgeon’s and scheduler’s prior judgment (e.g., their estimate), most variability in case durations contributing to lower productivity and reduced productivity of surgeons from late starts is caused by those cases that are of uncommon procedures [6,7]. Hospitals should exercise caution when considering the implementation of case-duration predictive models not tested on data that includes every performed case, rather than examining one or a few procedure categories, such as lower-extremity joint arthroplasty [6,7]. Hybrid methods can be used, incorporating data-intensive machine learning for common procedures and Bayesian methods for uncommon.

Organizational cautions in implementing machine learning and Bayesian methods

If an organization does not choose the hours into which cases are scheduled, "allocated time," based on maximizing the expected efficiency of use of operating room or anesthesia time, then more accurate case duration prediction generally will cause reduced operating room and anesthesia production (e.g., daily completed cases) [3,4]. The reason is that, in isolation, more accurate case-duration prediction results in long-duration cases being scheduled on future dates because they do not fit into earlier available operating room time [3].

Tuning and selecting machine learning methods to minimize the mean absolute error in case durations, or the mean absolute percentage error, will also reduce operating room and anesthesia productivity [5]. The reason is that good operating room control desk decisions to increase productivity cause increased variability in case durations (e.g., preferentially assigning the fastest pairing of anesthesiologists to surgeons in rooms expected to have overutilized time and trainees to rooms expected to have underutilized time) [5].

For organizations aiming to achieve on-time starts for to-follow surgeons, the managerially sound strategy is not to add first-case-of-the-day starts, which can reduce anesthesia productivity [39]. Rather, apply a Bayesian method for estimation of the mean case duration when scheduling the case [29-31]. Then, schedule those cases from the start of the day "down" and the end of the allocated time "up," with a maximum gap of one hour between surgeons [40]. Furthermore, for each set of three or four rooms fully scheduled for the day, plan an extra shared room to increase productivity and reduce tardiness, without increasing anesthesia costs [41-44]. Surgeon blocks and the master surgical schedule need to be planned to ensure surgeons operating on the same day can share the extra room when it is not occupied by urgent cases [44,45].

Freestanding ambulatory surgery centers and hospital outpatient departments differ

At least with respect to rare types of procedures, there have been no indications of convergence between freestanding ambulatory surgery centers and hospital outpatient departments. Furthermore, comparing Figure 1 panels A, B, and Figure 2 panels A, B with Figure 3 panels A, B, and Figure 4 panels A, B, hospital outpatient departments have not seen a progressive increase in the frequency of singletons and doubletons to match that of inpatient surgical suites. Rather, the frequency of uncommon Current Procedural Terminology codes has decreased somewhat in hospital outpatient departments over time. Collectively, the figures show that, at the statewide level and longitudinally, the three types of surgical suites have remained fundamentally distinct in terms of their case duration predictions, frequencies of rare procedures, and likely associated surgeon preference cards.

Limitations

The study was limited to one US state, although populous. On the other hand, the absence of other statewide or provincial data for comparison underscores precisely why our study was important. The previous large population study that motivated our work was conducted using the National Survey of Ambulatory Surgery more than 25 years ago, and that survey was discontinued in 2006 [11-13]. Comparisons cannot be made with the National Inpatient Sample or the Nationwide Readmissions Database because they lack dates beyond the year and quarter. Evaluating those databases for combinations of procedures is useful, but not for case duration; rather, for addressing different questions about the potential to forecast or understand surgical costs, as they involve every procedure performed during a hospitalization [8,9].

Our study was limited to case scheduling before the day of surgery. Even at the start of surgical closure, the specific surgical procedure matters because different procedures take different amounts of time to complete [36]. Nevertheless, the incremental value of intraoperative events derived from the electronic anesthesia real-time information system increases over time (e.g., changes in inspired volatile agent concentrations and fresh gas flows) [45]. We did not investigate the relevance of singletons and doubletons for estimating the times remaining in cases [31,46].

We defined procedures as unordered combinations of codes, in part because the state of Florida administrative data reported performed procedures, rather than scheduled procedures for which the sequence can be informative. There were two or more procedures for 41.2% of ambulatory combinations (Table 1) and 64.4% of inpatient combinations (Table 3), for a pooled average of 52.4%, where 52.4% = ({5,106,524 - 3,001,073} + {4,772,566 - 1,700,781})/(5,106,524 + 4,772,566). Defining these procedures as ordered sequences (e.g., procedure A then B versus B then A) would necessarily increase the prevalence of singletons and doubletons. Therefore, our results deliberately minimized the fractions of procedures considered singletons or doubletons.

## Conclusions

Many surgical procedures are performed rarely, resulting in little procedure-specific historical case duration data. Freestanding ambulatory surgery centers have, for >10 years, remained distinct from hospitals in performing more common procedures. Hospitals should plan for case scheduling so that approximately 84% of distinct combinations of procedures and 44% of cases will have little to no historical data, and even less so by the combination of procedure and surgeon. If developing machine learning models for prediction of operating room times, closure times, etc., investigators and potential users should incorporate Bayesian techniques for cases with little or no historical data, because these are the characteristics of most procedures, most lists of cases, and cause the greatest variability in decisions and tardiness experienced by the surgeons without a first case of the day start.
